# Prevalence of Adverse Childhood Experiences in Adolescents with Special Educational and Care Needs in the Netherlands: A Case-File Study of Three Special Educational and Care Settings

**DOI:** 10.1007/s40653-024-00613-w

**Published:** 2024-02-08

**Authors:** Gabriëlle Mercera, Jessica Vervoort-Schel, Evelyne Offerman, Sanne Pronk, Inge Wissink, Ramón Lindauer

**Affiliations:** 1 Koraal Center of Expertise, De Hondsberg, Hondsberg 5, Oisterwijk, 5062 JT The Netherlands; 2 Department of Psychiatry and Neuropsychology, Maastricht University, Vijverdalseweg 1, Maastricht, 6226 NB The Netherlands; 3 Department of Child Development and Education, University of Amsterdam, Nieuwe Achtergracht 127, Amsterdam, 1018 WS The Netherlands; 4 Orion, Special Education, Bijlmerdreef 1289-2, Amsterdam 1103 TV The Netherlands; 5 Academic Workplace Youth at Risk (AWRJ), Child and Adolescent Psychiatry & Psychosocial Care, Amsterdam UMC, Vrije Universiteit Amsterdam, Amsterdam, The Netherlands; 6 Department of Clinical Child & Family Studies, Utrecht University, Heidelberglaan 1, Utrecht, 3584 CS The Netherlands; 7 Levvel, Academic Center for Child and Adolescent Psychiatry, Meibergdreef 5, Amsterdam, 1105 AZ The Netherlands; 8 Department of Child and Adolescent Psychiatry, Amsterdam University Medical Centre, University of Amsterdam, Meibergdreef 5, Amsterdam, 1105 AZ The Netherlands

**Keywords:** Adverse childhood experiences, Behavioral problems, Special education, Youth care, Alternative education, Family context

## Abstract

To date, Adverse Childhood Experiences (ACEs) in adolescents with special educational and care needs have received little attention as an important risk factor for their behavioral, emotional, and learning problems. This study provides insight into ACE prevalence and family risk factors in three Dutch special educational and care settings for vulnerable school-aged youth. 268 adolescents (10–18 years old) with severe and persistent problems at individual and family level, from a special educational setting (setting 1; *n* = 59), a residential care setting (setting 2; *n* = 86) and an alternative educational setting (setting 3; *n* = 123) were included. A retrospective cross-sectional study design was used. Data were collected between 2016 and 2019 through structured case-file analysis. A substantial proportion of the adolescents in all settings experienced at least one ACE, with 69.5% in setting 1, 84.9% in setting 2 and 95.1% in setting 3. Family risk factors were relatively common, among which a limited social network in all settings (20–50%) and debts in setting 2 and 3 (25–40%). The substantial ACE prevalence underlines the need for early ACE awareness. Trauma-informed care and education are needed to adequately understand trauma-related behaviors, prevent retraumatization, and enhance learning and healthy development. Given that ACEs regarding household dysfunction and family risk factors seem to be common in adolescents with special educational and care needs, family centered approaches should be implemented as well in the interest of lifelong health and well-being for both adolescents and their families.

## Introduction

In recent decades, both research and practice have become more aware of the prevalence and impact of Adverse Childhood Experiences (ACEs). Historically, studies of ACEs focused on adults and despite an increased focus on children in ACEs research in recent years (Narayan et al., 2021), research on the prevalence of ACEs in children is still sparse. Moreover, children with special educational or care needs are rarely included (Massetti et al., 2020), while limited research shows that ACEs are associated with special educational and health care needs (Kan et al., 2020). There is an association between ACEs and problems that children with special educational and care needs face, such as developmental problems, learning difficulties, behavioral problems, social problems, health-risk behaviors, psychopathology, and mental and physical health problems (Bright et al., 2016; Garrido et al., 2018; Goldenson et al., 2020; Hunt et al., 2017; Liming & Grube, 2018; Schäfer et al., 2022). Moreover, ACEs are known to be negatively related to school success, such as poor academic performance, a lack of school engagement, reduced attendance at school and an increased risk for school dropout (Crouch et al., 2019; Morrow & Villodas, 2018; Stempel et al., 2017; Webster, 2022). More knowledge on the prevalence of ACEs in children with special educational and care needs is crucial to understand their problems and to improve learning, development and health outcomes. Therefore, this study aims to gain a better understanding of the prevalence of ACEs and other family risk factors among Dutch adolescents with special educational and care needs.

### Children with Special Educational and Care Needs

Children with special educational and care needs are characterized by their increased use of multiple systems of care (Kan et al., 2020). In a sample of children in the United States, aged 6–17, Kan et al. (2020) demonstrated that each additional ACE increased the likelihood of the child having a special educational or health care need by 26%, such as the need or use of specialized treatment, counselling for an emotional, behavioral or developmental condition and above-routine need of medical, mental health or educational services. When regular educational systems and primary care facilities can no longer adequately support the development of the child and the family, more intensive support is required. In The Netherlands there are various forms of more intensive youth care and educational services, among which special education, outpatient youth care services (e.g. outpatient care, day treatment, specialized treatment), residential youth care services, youth protection and probation measures, or a combination of these forms (Centraal Bureau voor de Statistiek, 2023; Nederlands Jeugdinstituut, *s.d.*). Children are eligible for this support if they are referred by, for example, a general practitioner, medical doctor, judge, youth protection service or the municipality. Thus by ‘children with special educational and care needs’ we refer to children who receive these more intensive forms of education or care than the regular education and primary care system in the Netherlands provides. When there are problems in multiple areas of life, such as psychological, physical or intellectual functioning, parenting, finances, housing or education, these forms of youth care provide assistance. Recently a Dutch study on the characteristics of youth receiving these forms of youth care services has been published (Centraal Bureau voor de Statistiek, 2023). Children who received youth care were more likely to attend special education, grow up in low-income or indebted households, or in households where someone uses mental health services and/or has an indication for intellectual disability support. For a better understanding of the potential role of ACEs in the challenges these children and families face, more knowledge on the prevalence of ACEs in children with special educational and care needs is needed. Given the research sample of the current study, we specifically focus on adolescents aged 12–18 years old.

### The Adverse Childhood Experiences Framework

ACEs are risk factors for development, health and societal functioning that are common, interrelated, and have cumulative effects (Gervin et al., 2022; Turney, 2020). Originally ACEs were defined as 10 experiences of abuse, neglect in the family or household dysfunction that significantly contribute to negative health outcomes in childhood, adolescence and/or adulthood (Anda et al., 2009; Felitti et al., 2019). These experiences encompass physical and emotional abuse, physical and emotional neglect, sexual abuse, parental separation or divorce, domestic violence against the mother, substance abuse of a household member, household member with mental illness and incarceration of a household member and are also referred to as ‘original ACEs’. The ACE framework continues to evolve. In recent years, other experiences have been recommended to be added to the ACE framework, such as peer victimization, battering, gambling problems in the household, contact with child protective services or foster care placement, poverty, neighbourhood violence or discrimination (Afifi et al., 2020; Hawes et al., 2021). Thus, the ACEs framework continues to expand with experiences outside the family unit, in the community (Hamby et al., 2021; Portwood et al., 2021). Given the still evolving developments in this area of research, there is not yet an internationally agreed definition of ACEs or a uniform operationalization to assess ACEs in children and adults (Karatekin & Hill, 2019). However, the original framework consisting of 10 ACEs is a worldwide commonly used and investigated operationalization and is therefore used in this study (Anda et al., 2009; Felitti et al., 2019).

### ACE Prevalence in children and adolescents

Prevalence findings on ACEs in children and adolescents that are available show considerable variation between studies due to for example different study populations, ACE frameworks, instruments and approaches and small sample sizes (Massetti et al., 2020). Therefore, when examining study results on ACE prevalence, these limitations of existing ACE research should be taken into account.

The systematic review by Carlson et al. (2020) found that approximately two-thirds of school-aged youth (i.e. < 18 years old) in the general population had had at least 1 ACE of the original framework, regardless of where they lived in the world. Most studies on ACE prevalence in adolescents have been conducted in the United States. A study among high school adolescents in the United States showed that 62.5% experienced at least 1 ACE of the original framework (Meeker et al., 2021). Another study in the United States using the original framework demonstrated an ACE prevalence of 55.7% among adolescents aged 12–17 (Bethell et al., 2017). In the Netherlands no studies specifically focusing on adolescents have been conducted. Dutch data on ACE prevalence in 9–13 year old children in regular elementary schools revealed that about 45% experienced at least 1 ACE and about 11% experienced 3 or more ACEs of 10 measured ACEs of the original framework (Vink et al., 2019).

There are limited findings available on the prevalence of ACEs in adolescents with special educational and care needs, and most studies on this vulnerable group include a broader age range. For example, the results of a nationally representative longitudinal study in the United States on children involved with child protection services found that by age 6, approximately 70% of the children had experienced 3 or more out of 8 ACEs of the original ACE framework (Clarkson Freeman, 2014). Pooled data of a systematic review showed that nearly 87% of justice-involved youth (i.e. < 18 years old) in 13 countries had experienced at least 1 ACE from the original framework, and that they were 12 times more likely than their peers to have experienced at least 1 ACE (Malvaso et al., 2021). Recent Dutch research among children with severe social-emotional and behavioral problems in primary and secondary special education schools aged 8–18 years showed that about 80% experienced at least 1 ACE and about 24% experienced 4 or more ACEs of the original ACE framework (Offerman & Asselman et al., 2022). Additionally, results of a Dutch study among children with cognitive and adaptive limitations receiving residential care revealed that approximately 86% of these children had experienced at least 1 ACE and 25% experienced 4 or more ACEs out of 10 ACEs from the original framework (Felitti et al., 2019; Vervoort-Schel et al., 2021). In sum, limited research has been done on ACEs in adolescents with special education and care needs and existing results are difficult to compare. All cited literature above is American or Dutch. The American youth care system cannot be compared one on one with the system in The Netherlands, due to for example differences in cultural contexts, accessibility of care and thus heterogeneity in populations. Yet, the available American and Dutch results do indicate an increased ACE prevalence amongst children and adolescents with special educational and care needs compared to studies in regular schools or based on studies on children and adolescents in the general population.

### Impact of ACEs on Child and Family Functioning

ACEs can derail neurodevelopment, especially during critical periods of brain plasticity such as the first years of life and adolescence (Bundy et al., 2018). The effects on emotional, social, behavioral, cognitive, mental and physical health can be detrimental (Morris et al., 2021). Studies have shown that adolescents who reported ACEs were more likely to experience depression, anxiety, drug abuse, antisocial behavior, suicidality and cognitive difficulties (Meeker et al., 2021; Olofson et al., 2018; Schilling et al., 2007). Ensuring safe, stable, nurturing relationships for all children and adolescents in all settings is key to preventing ACEs and mitigating their effects in the interest of lifelong health and the health and well-being of future generations (Gervin et al., 2022; Merrick et al., 2020). Unfortunately, family stressors such as debts, housing problems, a limited social network, parental intellectual disabilities, or parental ACEs can compromise safe, stable, nurturing relationships and be a risk factor for parenting and family functioning (Crouch et al., 2019; May & Harris, 2020; Merrick & Guinn, 2018; Thornberry et al., 2013; Vervoort-Schel et al., 2021). Since a wide range of family risk factors can impact child and family functioning, the current study focuses on family risk factors as well, besides its focus on the ACEs from the original framework.

### Objectives of This Study

To date, for adolescents with special educational and care needs, ACEs have been overlooked as an important risk factor for behavioral, emotional, and learning problems and the opportunities for them to optimally benefit from or prevent special education and youth care. Therefore, this study gives insight into the prevalence of ACEs and family risk factors in a convenience sample of adolescents with special educational and care needs from three specialized educational and youth care settings in The Netherlands. The original ACE framework (Anda et al., 2009; Felitti et al., 2019), consisting of ten ACEs, was used in this study. For these ACEs a similar operationalization was available between the three settings. In addition, six family risk factors were included, since these factors are known to impede safe, stable, nurturing relationships and thereby family functioning.

The current study specifically focuses on adolescents. The settings include: (1) two special education schools; (2) a residential youth care center and (3) an outpatient alternative educational facility providing both special education and youth care. Thereby, our convenience sample consists of three of the four categories of more intensive forms of youth care services that are provided in The Netherlands as described in this introduction. These settings are indicated when regular education systems and primary care facilities can no longer adequately support the development of the child and the family. Adolescents in all three settings experience a combination of severe and persistent individual, family, and social context problems, but the settings respond to different needs: special education only, residential admission or a combination of special education and youth care services from day admission (see the paragraph ‘settings’ for a detailed description). Therefore the prevalence of ACEs and family risk factors are explored per setting. This study provides a unique insight into ACE prevalence in three underrepresented vulnerable subgroups of adolescents with special educational and care needs. This study aims to contribute to a better understanding of the potential role of ACEs and family risk factors in the challenges these adolescents face, and to inform research, policy an practice.

## Methods

### Design and Procedures

This study is based on a convenience sample which stems from three independent larger studies investigating ACEs in vulnerable school-aged populations, which were conducted at schools from a special education foundation (Offerman et al., 2022), a national residential youth care center (Vervoort-Schel et al., 2021), and an alternative educational facility (Pronk et al., 2020). All studies used a retrospective cross-sectional study design and the variables were selected based on scientific literature on ACEs and family risk factors. All variables were operationalized in codebooks. Data were collected between 2016 and 2019 by means of structured analysis of case-files, containing reports such as school, youth care, diagnostic, and psychiatric reports from previous and current schools or care settings, as well as day-to-day journals at two of the three settings (i.e., the special education schools and the alternative educational facility). The reports included information about the adolescent, their parents and family and social context. For the present study, we combined the fully anonymized data of the three separate studies. Each separate study protocol was approved by the Ethics Review Board of the University of Amsterdam (residential care center 2018-CDE-8871; special educational setting 2017-CDE-7603; alternative educational facility 2017-CDE-7736).

### Setting

The sample consisted of Dutch adolescents who had been assigned to special education (setting 1), a residential youth care center (setting 2) or an alternative educational facility (setting 3).

**Setting 1.** Two urban secondary special education schools for students with emotional and behavioral disorders were included. These special education schools provide education, care and guidance for youth between 11 and 18 years old (by exception from 10 years old) with severe and persistent internalizing and/or externalizing behavioral and social emotional problems, whose educational and support needs could not sufficiently be met by regular schools. Previous research within this setting explored 172 case-files of 8 to 18 year old students with emotional and behavioral disorders and demonstrates for example that about 68% of the students had two or more school switches prior to their special education placement at the time of the case-file analysis. The number of school switches in this population is corrected for the primary to secondary school switch. Autism spectrum disorders (35%), attention-deficit/hyperactivity disorder (35%) and oppositional defiant disorder (17%) are the most commonly reported diagnoses, with 41% of the students having comorbid classifications of the 4th or 5th edition of The Diagnostic and Statistical Manual of Mental Disorders (DSM-4; DSM-5; Americal Psychiatric Association, 2000; American Psychiatric Association, 2013). 54% of the students used medication related to their DSM-4 or DSM-5 classifications. 86% of the students had received family-oriented social work and therapy, and 87% had received a form of therapy focussing on the child’s individual problems (Offerman et al., 2022). Often, a combination of child, family and school factors plays a role in special education placement. Mostly, this placement is voluntary – as a last opportunity to receive education – after an accumulation of negative experiences at previous, often regular schools. Youth participate in the special education program during the hours of a regular school day (i.e., approximately six hours per day), potentially at least up until they reach the compulsory school age of 16 years old. Apart from special education, various specialized youth health care partners provide diagnostics and individual guidance or treatment in the schools in collaboration with the school and parents, to reduce problem behavior and enhance cognitive and social emotional development.

**Setting 2.** One national residential youth care center situated in a rural area in the Netherlands was included. This setting provides specialized clinical observation, diagnostics and treatment for children between 2 and 18 years old, with intellectual disabilities and borderline intellectual functioning and severe, persistent and complex mental- and behavioral health problems. Previous research within this setting, exploring 69 case-files of children and adolescents (≤ 18 years old) with intellectual disabilities, gives an indication of the problems those children and adolescents face (Vervoort-Schel et al., 2018). Outcomes of the Child Behavior Checklist (CBCL; Achenbach & Rescorla, 2001), reported by residential care staff, showed that almost two third of youth scored in the borderline clinical or clinical range for total problem behavior and around half of the children scored in the borderline clinical or clinical range for externalizing behavior problems and internalizing behavior problems. One third of the sample experienced attachment problems and/or trauma- and stressor-related problems. Another study within this setting, exploring 134 case-files of children and adolescents with intellectual disabilities or borderline intellectual functioning, showed that 85% of the children had a co-morbid clinical disorder (Vervoort-Schel et al., 2021). In this study it was also found that family problems were relatively often present, as 76% of the children grew up in families with multiple and complex problems. These are families experiencing an accumulation of problems in at least six out of the following seven domains: child, parental, child rearing, family functioning and contextual factors, social network and prior history of support services (Dekovic & Bodden, 2019). About 28% of the children had parents with intellectual disabilities, about 32% parents with ACEs and 28% had parents with debts (i.e. meaning that a professional reported in the case-file that the family in which the child grew up experienced any kind of debts or that debt counselling agencies have been involved). The residential placement is mostly voluntary, however, in some cases placement is ordered by court. Youth participate in a 24-hour program for approximately 1.5 years. During placement, the great majority of the included adolescents participate in a program of the involved special educational school for students with intellectual disabilities.

**Setting 3.** One urban alternative educational facility was included. This facility provides education and care for youth between 12 and 18 years old, who are at risk for school drop-out or (secure) residential placement because of a complex combination of individual, family, and social context problems. This facility was developed in 2011 as an innovative program to reduce the number of residentially placed youth, especially in secure residential facilities. Participation in the program can be voluntary as well as stimulated or ordered by court as a last chance to avoid secure residential placement. Youth participate in the 12-hour program (i.e., 8 a.m. to 8 p.m. program), for around six to nine months, before returning to regular or special education, after which their coach stays involved for at least six more months. The program is an integration of special education and youth care (i.e., academic classes, individual and group trainings, mental health therapies, workshops) and focuses on all life domains (i.e., school, home, leisure time). Previous research based on case-files gives an indication of the severity of problems of adolescents at this alternative educational facility. Adolescents of the studied facility for example face limited intellectual abilities (33%), trauma- and stressor-related disorders (15%), disruptive behavior disorders (41%), substance use disorders (16%), and criminal involvement (20%), based on case-file analysis (Pronk et al., 2020). Furthermore, based on structured questionnaires (Pronk et al., 2021), adolescents score in the borderline clinical or clinical range on the Brief Problem Monitor (BPM; Achenbach et al., 2011) for externalizing behavior problems for 83% (teacher-reports), 40% (parents-reports) and 13% (self-reports) and for internalizing behavior problems 48% (teacher-reports), 37% (parents-reports) and 10% (self-reports). Furthermore, 64% of parents reported severe parenting stress, and 43% mild or severe problems in family functioning. Psychologists reported severe to extreme problems on adolescent functioning (57%), quality of the context (72%), severity of needs (56%), and urgency of needs (71%).

### Sample

The combined sample consisted of 268 adolescents (*N* = 268), between 10 and 18 years old (*M*_age_ = 14.2 years; 66% boys); 59 from setting 1 (special educational schools); 86 from setting 2 who were discharged between 2016 and 2019 (residential care center); 123 from setting 3 who were assigned to the setting between 2014 and 2017 (alternative educational facility). For this study, only the subgroup of adolescents from 12 to 18 of setting 1 and 2 were included. All were assigned to a special educational or care setting - hereafter referred to as ‘setting’ - due to severe and persistent problems at the individual, family and context level.

Background information on the behavioral problems of the adolescents was present from the samples of setting 1 and 2. In these settings the outcomes of the Brief Problem Monitor (BPM; Achenbach et al., 2011; setting 1) and the Child Behavior Checklist/6–18 (CBCL; Achenbach & Rescorla, 2001; setting 2), reported by parents/caregivers, were collected. In setting 3 this data was not available. Clinical scores on internalizing problems were present in 13.6% of the adolescents in setting 1 and in 47.7% of the adolescents in setting 2. Clinical scores on externalizing problems were present in 28.8% of the adolescents in setting 1 and in 53.5% of the adolescents in setting 2.

### Measures

To systematically collect the information for the current study, case-files were assessed using scoring protocols and codebooks, available from the authors. The operationalizations used in each study were extensively compared and for some variables a minor recoding was done on the original data to guarantee conformity (e.g., the categorization of country of birth of parents). The following operationalizations were used:

**Demographic characteristics.** Four items were included: gender, age at the start of the intervention, age during case-file study or after intervention, and the country of birth of parents.

**Adverse Childhood Experiences.** Ten variables were assessed, based on the original ACE framework (Anda et al., 2009; Felitti et al., 2019), that is physical abuse, emotional abuse, physical neglect, emotional neglect, sexual abuse, parental incarceration, parental separation or divorce, domestic violence, parental substance abuse and parental mental health problems. These items were coded based on information derived from a professional report in one or more documents in the case-file. ACEs were coded as present (1) if the description in the data-files matched the chosen operationalization. If the information did not match, the ACE was coded as absent (0). Table 1 presents the ACEs used with accompanying definitions. The sum of the 10 original ACEs was calculated, resulting in a total score (0 to 10).

**Table 1:** Operationalization of adverse childhood experiences based on the original framework (Anda et al., 2009)

Physical abuse	The child experienced pushing / beating / grabbing / slapping / kicking or being hit so hard by (one of) the biological parents or primary caregiver(s) that it resulted in marks or injury
Emotional abuse	The child was sworn at / insulted / threatened or put down by (one of) the biological parents or primary caregiver(s) that may physically have hurt the child
Physical neglect	The parent’s or primary caregiver’s behavior interfered with the child’s care; wearing dirty clothes / bad hygiene / not enough personal living space / no safe living space / not enough to eat / not taken to a doctor when needed or forced to take care of themselves
Emotional neglect	The parent(s) or primary caregiver(s) didn’t make the child feel special and loved / the family not being a source of strength, protection and support or the child received little attention
Sexual abuse	The child was involuntarily touched in a sexual way / forced into any form of sexual contact / forced into watching sexual content by a parent(s), primary caregiver(s), adult, relative, family friend or stranger
Parental incarceration	A parent or primary caregiver went to prison
Parental separation or divorce	The biological parents were (temporary) separated or divorced
Domestic violence	The father, mother or primary caregiver was (1) pushed, grabbed, slapped, or had something thrown at her/him (2) kicked, bitten, hit with a fist, or hit with something hard (3) repeatedly hit over at least a few minutes (4) threatened with or hurt by a knife or gun
Parental substance abuse	The parent or primary caregiver used excessive alcohol or drugs
Parental mental health problems	1) biological parent(s) had mental health problems (symptoms or disorders) interfering with the child’s care 2) a parent ever attempted suicide

**Family risk factors.** Six items were included, partially based on expanded ACE frameworks (Crouch et al., 2019; Jaffee et al., 2013; Merrick & Guinn, 2018; Thornberry et al., 2013; Vervoort-Schel et al., 2021). These items were residential care placement, debts, housing problems, intellectual disabilities of the parent(s), ACEs of parents and a limited social network. ACEs of parents were coded in setting 1 and 2 only. In the residential care center, the variable residential care placement concerned the presence of an out of home placement before admission to the current residential care setting. Table 2 presents the family risk factors with accompanying definitions.

**Table 2:** Operationalization of family risk variables

Intellectual disabilities parents	There was a (suspicion of) intellectual disability of (one of) the biological parents
ACEs parent	Parent(s) experienced at least one of ten ACEs*
Debts	The family experience(d) economic hardship (e.g., financial problems, debts)
Housing problems	The family experience(d) housing problems
Limited social network	The family has a limited social network
Residential care placement	The child was separated from parents due to out-of-home placement

* According to the original framework (Anda et al., 2009)

Each study of the three different settings had an inter-rater reliability percentage which can be considered as sufficient. In setting 1 and 2, the percent agreement was calculated. This statistic is directly interpreted as the percent of data that are correct (McHugh, 2012). In setting 3 the Cohen’s Kappa was calculated which accounts for false agreements. For 10.5% of the case-files in setting 1, the inter-rater reliability for the complete codebook (162 items), including the ACEs, was 86.4%. For 25.0% of the case-files of the total case-file study (*N* = 169) in setting 2, the inter-rater reliability was 96.6%. For 11.0% of the case-files of setting 3, the Cohen’s Kappa for the ACEs was 0.82 which is considered as good (McHugh, 2012). Due to initial low inter-rater reliability scores for some categories in this setting, additional agreements were made and another 11.0% of case-files resulted in 68.0% for the subscale emotional neglect (moderate) and 35.0% for the subscale physical neglect (minimal). Therefore, for these two categories, every case-file was discussed and scored together to reach consensus.

### Statistical Analyses

All statistical analyses were conducted in SPSS, version 26 (IBM, Armonk, NY, USA). Descriptive statistics were used to present the demographics and to explore the prevalence of ACEs and variables in the family context in the total sample and by setting.

## Results

In Table 3 the demographic data of the sample are presented. The mean age (start) and the mean age (at the time of case-file review/after intervention) in the total sample was respectively 14.2 (range 10–18) and 15.3 years old (range 12–19). Differences between settings in age, gender and country of birth of parents were explored to understand how the settings relate to each other in terms of demographics. There were significant differences between the groups in age (start) and age (at time of case-file review/after intervention). Post hoc comparisons indicated that both age variables of the adolescents in the alternative educational facility (setting 3) were higher than the age variables of the adolescents in special education (setting 1) and in the residential care center (setting 2). In all settings, the study sample consisted of more males than females. Gender differences between groups were found, in which the proportion males was significantly higher in setting 1 than in setting 2 and setting 3. Although there were differences between settings in age variables and gender, logistic regression analyses showed that these demographic variables did not significantly impact the number of ACEs in these settings (setting 1: *X*^2^(3, *n* = 59) = 0.46, *p* > .05; setting 2: *X*^2^(3, *n* = 86) = 0.68, *p* > .05; setting 3: *X*^2^(3, *n* = 123) = 0.90, *p* > .05). As assumptions for the variable Country of birth of parents were violated, a Chi-square test was performed for a combined group of categorizations, that is, (1) both parents born in the Netherlands and (2) at least one parent born in a foreign country. It was found that the proportion of both parents born in the Netherlands differed significantly between all three settings, with the highest proportion of both parents born in the Netherlands in setting 2. Also, the proportion of at least one parent born in a foreign country differed significantly between all three settings, with the highest proportion of at least one parent born in a foreign country in setting 3.

**Table 3:** Demographics

	Setting 1: Special education (*n* = 59)	Setting 2: Residential care center (*n* = 86)	Setting 3: Alternative educational facility (*n* = 123)	Total (*n* = 268)	*p*
Gender*					
Male	86.4% (51)_a_	58.1% (50)_b_	61.8% (76)_b_	66% (177)	*X*^*2*^ (2) = 14.335, *p* = .001
Female	13.6% (8)_a_	41.9% (36)_b_	38.2% (47)_b_	34% (91)	
Age at start intervention (*M*)*	13.3_a_ SD 1.4 RNG 11–17	13.3_a_ SD 1.7 RNG 10–17	15.3_b_ SD 1.1 RNG 12–18	14.2 SD 1.7 RNG 10–18	*H* (2) = 92.803, *p* = .000
Age during case-file review / after intervention (*M*)*	14.7_a_ SD 1.6 RNG 12–19	14.5_a_ SD 1.8 RNG 12–19	16.0 _b_ SD 1.2 RNG 12–18	15.3 SD 1.6 RNG 12–19	*H* (2) = 50.703, *p* = .000
Country of birth of parents*					
Both parents born in The Netherlands	27.1% (16)_a_	65.1% (56) _b_	14.6% (18)_c_	33.6% (90)	*X*^*2*^ (2) = 56.824, *p* = .000
At least one parent born in a foreign country	61.0% (36)_a_	29.1% (25)_b_	74.8% (92)_c_	57.1% (153)	

*significant Difference(s) between Groups

_a, b, c_ Within each row, percentages that significantly differ between settings at the 0.05 level get different subscript letters (i.e. ‘a’ differs from ‘b’; ‘a’ and ‘a’ both differ from ‘b’; ‘a’, ‘b’, ‘c’ all differ from eachother; no subscript letter indicates no significant difference between the setting and other settings)

Table 4 presents the prevalence of ACEs. A substantial proportion of the adolescents in all settings experienced at least 1 ACE, with 69.5% in setting 1, 84.9% in setting 2 and 95.1% in setting 3. In setting 2, even 81.3% of the adolescents experienced at least 2 ACEs and 62.6% at least 3 ACEs. The proportion of at least 2 ACEs in setting 1 and 2 was respectively 35.6% and 58.1%. The proportion of at least 3 ACEs in setting 1 and 2 was respectively 23.7% and 48.8%.

The mean number of ACEs in the total sample was 2.7, with a relatively high standard deviation (*SD* = 2.0) and large range (0–9), see Table 4 as well. In setting 1, 2 and 3 the mean number of ACEs was respectively 1.6 (*SD* = 1.9; range 0–8), 2.4 (*SD* = 1.8; range 0–9) and 3.3 (*SD* = 2.0; range 0–9).

Exploring the prevalence of the types of ACEs, it was found that parental separation or divorce was most prevalent in all settings, namely 47.5% in setting 1, 59.3% in setting 2 and 74.8% in setting 3. Emotional neglect was especially present in setting 3 (61.8%). Yet, in setting 1 and 2 the presence of this ACE was relatively high as well (approximately one-fifth of the adolescents) compared to other types of ACEs within these settings. In all settings approximately 30–40% of the biological parents experienced psychological problems. Domestic violence was relatively common among adolescents in setting 2 (26.7%) and setting 3 (28.5%). In all settings less common ACEs were incarceration of parents or primary caregivers and sexual abuse.

**Table 4:** Prevalence of adverse childhood experiences

	Setting 1: Special education (*n* = 59)	Setting 2: Residential care center (*n* = 86)	Setting 3: Alternative educational facility (*n* = 123)	Total (*n* = 268)	
*Minimum number of ACEs*					
≥ 1 ACEs	69.5% (41)	84.9% (73)	95.1% (117)	86.2% (231)	
≥ 2 ACEs	35.6% (21)	58.1% (50)	81.3% (100)	63.8% (171)	
≥ 3 ACEs	23.7% (14)	48.8% (42)	62.6% (77)	49.6% (133)	
≥ 4 ACEs	15.3% (9)	24.4% (21)	42.3% (52)	30.6% (82)	
≥ 5 ACEs	8.5% (5)	10.5% (9)	22.0% (27)	15.3% (41)	
≥ 6 ACEs	6.8% (4)	4.7% (4)	15.4% (19)	10.1% (27)	
**Number of ACEs**	1.6 SD 1.9 RNG 0–8	2.4 SD 1.8 0–8 RNG	3.3 SD 2.0 RNG 0–9	2.7 SD 2.0 RNG 0–9	
*Type of ACEs*					
Physical abuse	13.6% (8)	18.6% (16)	31.7% (39)	23.5% (63)	
Emotional abuse	11.9% (7)	15.1% (13)	21.1% (26)	17.2% (46)	
Physical neglect	16.9% (10)	8.1% (7)	48.0% (59)	28.4% (76)	
Emotional neglect	22.0% (13)	23.3% (20)	61.8% (76)	40.7% (109)	
Sexual abuse	1.7% (1)	14% (12)	13% (16)	10.8% (29)	
Parental separation/divorce	47.5% (28)	59.3% (51)	74.8% (92)	63.8% (171)	
Psychological problems biological parent(s)	28.8% (17)	39.5% (34)	29.3% (36)	32.5% (87)	
Substance abuse parent/primary caregiver	6.8% (4)	22.1% (19)	19.5% (24)	17.5% (47)	
Domestic violence	11.9% (7)	26.7% (23)	28.5% (35)	24.3% (65)	
Incarceration parent/primary caregiver	3.4% (2)	9.3% (8)	5.7% (7)	*6.3% (17)*	

Table 5 describes the prevalence of family risk factors. In setting 1, a limited social network of the family was present in at least half of the adolescents. In setting 2 having a parent with a suspected intellectual disability, a family with debts and a family with a limited social network was prevalent in about a quarter of the adolescents. In setting 3, relatively many (42.3%) adolescents experienced an out-of-home placement in residential care. All adolescents in setting 2 were in residential care at that specific setting, but 44.2% of them already experienced out-of-home placements before the placement in setting 2.

**Table 5:** Family risk factors

	Setting 1: Special education (*n* = 59)	Setting 2: Residential care center (*n* = 86)	Setting 3: Alternative educational facility (*n* = 123)	Total (*n* = 268)
Intellectual disabilities parent(s) (suspicion)	0% (0)	25.6% (22)	9.8% (12)	12.7% (34)
ACEs parent(s)	16.9% (10)	22.1% (19)	-	10.8% (29)
Debts	8.5% (5)	23.3% (20)	39.8% (49)	27.6% (74)
Housing problems	5.1% (3)	7.0% (6)	19.5% (24)	12.3% (33)
Limited social network	52.5% (31)	26.7% (23)	20.3% (25)	29.5% (79)
Residential care placement	16.9% (10)	44.2% (38)	42.3% (52)	37.3% (100)

## Discussion

In the current study, we have explored the prevalence of ACEs and family risk factors in Dutch adolescents with special educational and care needs in three settings (i.e., special education, residential youth care and an alternative educational facility). The aim is to raise awareness of the prevalence of ACEs in this vulnerable group of adolescents and to advance research, policy and practice in this area. Two main findings are discussed below, which are (1) ACE prevalence is substantial in adolescents with special educational and care needs and (2) family risk factors are common in the overall sample.

First, it was found that a large proportion of the adolescents in all settings experienced at least 1 ACE, with 69.5% in setting 1, 84.9% in setting 2 and 95.1% in setting 3. At least 2 ACEs were experiences by 35.6%, 58.1% and 81.3% of the adolescent in respectively setting 1, 2 and 3. These prevalence rates are relatively high compared to ACE research in adolescents in the general population, as described in the introduction section of this study. These studies showed that the prevalence of at least 1 ACE was 56.0% (Bethell et al., 2017) and 62.5% (Meeker et al., 2021). Our prevalence rates are also relatively high compared to studies on children in a broader age range, in which the prevalence of at least 1 ACE varied from 45 to 66% and the prevalence of at least 3 ACEs ranged from 4 to 10% (Bright et al., 2016; Carlson et al., 2020; Turney, 2020; Vink et al., 2019). Although the mean age of the current study population was higher (*M* = 15.3) compared to the mean ages in for example the studies of Bright et al. (2016; *M* = 8.6 years) and Vink et al. (2019; *M* = 11 years), the ACE prevalence in the current sample of adolescents with special educational and care needs still seems considerable high, also in light of the finding of the present study that age was not significantly associated with the number of ACEs.

Our findings indicate a substantial ACE prevalence in adolescents with special education and care needs, which is consistent with national (Offerman & Asselman et al., 2022) and international (Clarkson Freeman, 2014; Malvaso et al., 2021) data on children and adolescents with special needs, also described in the introduction. All three settings in the current study provide education and/or care for children and adolescents with severe and persistent mental- and behavioral problems. As the number of ACEs increases, the risk for developmental, behavioral, emotional and learning problems and subsequent special education and care needs increases as well (Felitti et al., 2019; Kan et al., 2020). The cumulation of ACEs may underlie or contribute to the severe and persistent mental and behavioral problems for which the adolescents in the current sample receive special education and care. Since the relationship between ACEs and mental health and behavioral problems could not be investigated in the present study, it is not possible to draw conclusions about such associations. However, based on literature and the high prevalence of both mental health and behavioral problems and ACEs, a relationship between these factors seems plausible.

Second, besides ACEs, family risk factors were clearly present in the overall sample, with debts, limited social networks and previous residential care placements being the most prevalent. These family risk factors impair parental and family functioning, can induce the likelihood of experiencing ACEs and reduce the likelihood of safe and stable nurturing parent-child relationships (Schofield et al., 2018; Vervoort-Schel et al., 2021). There were also parents with ACEs or intellectual disabilities. It is known that parental ACEs, intellectual disabilities or psychological problems, the latter of which was a relatively common ACE in the current sample, can disrupt parental and family functioning and negatively affect their children’s outcomes (Crouch et al., 2019; Jaffee et al., 2013; May & Harris, 2020; Zhang et al., 2022). Kan et al. (2020) found that living with a family member with psychological problems was associated with twice the odds of the child having special educational and care needs. It was demonstrated that this association might be explained by the strong association between living with a family member with psychological problems and the child having an emotional or behavioral disorder (Kan et al., 2020). Vice versa, it is known that raising a child with mental health problems, can strain parenting and parental mental health and increase the risk for the development of ACEs experienced by children (Murphy, 2011). Thus, the health of the child and the health of parents are interconnected (Purpura et al., 2021) The consequences of ACEs and family risk factors on children’s development, behavior and learning could lead to increased needs that require more intensive forms of special educational and youth care services. These results illustrate the importance of safe, stable and nurturing environments and promoting resilience to buffer against the often present family adversities in adolescents with special education and care needs (Burstein et al., 2021). Future research should address the mechanisms through which ACEs and family risk factors affect special educational and care needs. This knowledge can help to mitigate the effects of ACEs and family risk factors in the interest of lifelong health and well-being of children and their families.

### Study Limitations and Strengths

Several limitations of this study should be considered. First, this study was based on a convenience sample with relatively small sample sizes of heterogeneous groups of adolescents in three different settings, experiencing a variety of severe and persistent and mental and behavioral problems, social emotional problems and/or intellectual disabilities or borderline intellectual functioning. Therefore, generalizing the results to other populations internationally with specific special education and care needs should be done with caution. However, this study is a unique contribution to the limited literature on ACEs in the broad population of adolescents with special needs, since it gives insight into three settings providing special education and/or care. Second, these descriptive data were retrieved from case-files in which potentially relevant information may not or may differently be administered by involved professionals. Inherent in this type of data is that there is little knowledge on accuracy and missings. Also, this makes it plausible that there is an underestimation of prevalence rates on ACEs and family risk factors. Third, no associations between ACEs and the mental and behavioral problems that were present (see for a description the methods section) in the study population could be explored, since the variables of the three settings regarding mental health and behavioral problems were not comparable due to different operationalizations. However, it is known that ACEs contribute to a variety of negative health outcomes (Felitti et al., 2019). As ACE research is limited in children and adolescents with special education and care needs, it is important to gain more insight into these associations in follow-up research. Fourth, as was stated in the introduction, the scope of the original ACE framework is limited, which could have led to underexposure of other adversities that may also negatively impact health outcomes. Finally, only ACEs and family risk factors were included in the present study, while protective and compensatory experiences (PCEs) are a powerful predictor of development and health as well, and can promote healthy outcomes and resilience (Burstein et al., 2021; Hays-Grudo & Morris, 2020). Children’s dependence on their environment is high and ensuring safe, stable, nurturing relationships for all children in all settings is key to preventing ACEs and mitigating their effects in the interest of lifelong health (Gervin et al., 2022). For future ACE research we recommend to examine protective and compensatory experiences as well to get a better understanding of developmental trajectories and health outcomes.

Notwithstanding its limitations, the present study has several strengths. First, since ACE research on youth with special care or educational needs is limited (Massetti et al., 2020), this study is a unique contribution to the insights on ACE prevalence in this vulnerable group. Second, the data collections and operationalizations of the variables in this study were structured and accurate, reflected in good to high inter-rater reliability scores in all three settings. Third, this study contributes to literature since it was the first to describe ACEs in adolescents in three different special needs/care settings, which is exceptional in ACE research since different frameworks are often being used.

### Implications

The high prevalence of ACEs in the current sample of adolescents with special educational and care needs underlines the need for increased ACE awareness in education, healthcare, policy and in society in general. When there is insufficient attention for ACEs and their detrimental impact on a wide variety of health outcomes such as social, emotional and cognitive development and subsequent mental and behavioral problems, there is a risk for treating symptoms instead of underlying root causes. By integrating trauma-informed approaches such as Trauma-informed care (TIC) or Trauma-informed education, in which past and present positive and adverse experiences are included to understand behavior, and in which professionals are supported to prevent retraumatization and provide adequate support, appropriate and sustainable education and care can be provided (Thirkle et al., 2021).

The finding that a significant part of the studied population already experienced at least 3 or 4 ACEs at the age of 15, outlines the need for recognition, prevention and early intervention of adverse experiences in children and adolescents. Throughout childhood, together with the first 1000 days in a child’s life, puberty constitutes the most active period for the development of different brain networks (Bundy et al., 2018). During both periods, brain development is the most vulnerable. Awareness of consequences of toxic stress resulting from ACEs in relation to brain development and neuroplasticity of key regions controlling cognitive processing and emotion regulation is crucial to stop the negative cycle of ACEs and its impact on overall health as early as possible (Weems et al., 2021).

The relatively large range in number of ACEs within settings, underlines that adolescents with special educational and care needs have different individual backgrounds. The emotional and behavioral problems of this vulnerable population are internationally increasingly seen as the result of complex interactions between child and environment (Köhne & Van Os, 2021; Lehman et al., 2017). This demonstrates that the personal life stories, backgrounds and individual educational and care needs of the child and family are an important source of information for personalized, effective and sustainable educational and health care trajectories. To what extent the differences in prevalence of ACEs and family risk factors between settings contributed to the allocation of the adolescents to the specific setting is not examined in the current study. Also, research on the impact of different types of ACEs on development is still ongoing (Miller et al., 2018). What is known is that ACE awareness takes an important place in working with children and adolescents with a developmental, emotional and behavioral problems or disrupted family functioning. Also in the case of relatively mild symptomatology or dysregulation a close look at the possible underlying presences of ACEs is important with the aim of minimizing long-term and intensive educational and care interventions.

Thus, ACEs are common in children and adolescents with special educational and care needs and therefore it is important to gain insight into possible experienced ACEs during admission and provision of education and/or care. ACE assessment is not yet common in special education and youth care in the Netherlands and there are some contemporary challenges, which call for awareness (Bartlett, 2020; McLennan et al., 2020). These challenges concern for example the uniformity of the framework and determinants to include (Bartlett, 2020; Finkelhor, 2018; Jee & Forkey, 2022; Karatekin & Hill, 2019), the necessary availability of effective interventions during and after screening (Finkelhor, 2018; McLennan et al., 2020) and the absence of valid screening tools (Jee & Forkey, 2022; Meehan et al., 2022). For example, when asking about ACEs professionals must be able to offer adequate interventions and responses to those with positive ACE screening, The right use of ACE screeners depends on the context in which professionals use it. Despite these challenges, having conversations about life history, sources of stress and strengths is key in education and care for both the child, the parents and the professional. Trauma-informed care and trauma-informed education can provide organizations a context to recognize and respond to ACEs in a trauma-sensitive way. Also, the use of a multi-informant perspective (i.e. child, family members, professional) seems important, as different informants can contribute to a more complete picture of the number, type and timing of ACEs (Hambrick et al., 2019; Offerman & Asselman, 2022; Zelechoski et al., 2021). Since ACEs and a lack of protective and compensatory experiences are often part of the child’s problems, this should be described in all personal plans and the education and care that is subsequently offered should be trauma-informed, with sensitivity to positive childhood experiences in children and parents (Kan et al., 2020; Narayan et al., 2021).

## Conclusion

To date there has been little scientific research on both ACE prevalence and family risk factors in adolescents with special educational and care needs. The current study contributes to limiting this knowledge gap by providing insight into the ACE prevalence of this vulnerable population. The substantial ACE prevalence in our sample of adolescents with special educational and care needs underline the need for early ACE awareness and a trauma-informed perspective in special educational and youth care interventions. When there is insufficient attention for ACEs and their detrimental impact on a wide variety of health outcomes, there is a risk for treating symptoms instead of underlying root causes. Approaches such as Trauma-informed care and Trauma-informed education should be impelemted in organizations working with these adolescents and their parents, in which past and present positive and adverse experiences of all involved are included to understand trauma-related behavior in adolescents and families (Thirkle et al., 2021). Such approaches can prevent retraumatization and support learning and healthy development in vulnerable groups of adolescents with special educational and care needs (Substance Abuse and Mental Health Services Administration [SAHMSA], 2014). In this, a family centered approach should be incorporated as well, given that ACEs regarding household dysfunction and family risk factors are common in adolescents with special educational and care needs. Future research should address the mechanisms through which ACEs and family risk factors affect special educational and care needs. This knowledge can help to mitigate the effects of ACEs and family risk factors in the interest of lifelong health and well-being of both adolescents and their families.
